# Identification of Nedd4 E3 Ubiquitin Ligase as a Binding Partner and Regulator of MAK-V Protein Kinase

**DOI:** 10.1371/journal.pone.0039505

**Published:** 2012-06-20

**Authors:** Svetlana V. Kalinichenko, Keiji Itoh, Elena V. Korobko, Sergei Y. Sokol, Vladimir L. Buchman, Igor V. Korobko

**Affiliations:** 1 Institute of Gene Biology, Russian Academy of Sciences, Moscow, Russia; 2 Department of Developmental and Regenerative Biology, Mount Sinai School of Medicine, New York, New York, United States of America; 3 Cardiff University, School of Biosciences, Cardiff, United Kingdom; University of Pittsburgh, United States of America

## Abstract

MAK-V/Hunk is a scantily characterized AMPK-like protein kinase. Recent findings identified MAK-V as a pro-survival and anti-apoptotic protein and revealed its role in embryonic development as well as in tumorigenesis and metastasis. However molecular mechanisms of MAK-V action and regulation of its activity remain largely unknown. We identified Nedd4 as an interaction partner for MAK-V protein kinase. However, this HECT-type E3 ubiquitin ligase is not involved in the control of MAK-V degradation by the ubiquitin-proteasome system that regulates MAK-V abundance in cells. However, Nedd4 in an ubiquitin ligase-independent manner rescued developmental defects in *Xenopus* embryos induced by MAK-V overexpression, suggesting physiological relevance of interaction between MAK-V and Nedd4. This identifies Nedd4 as the first known regulator of MAK-V function.

## Introduction

MAK-V/Hunk belongs to the group of AMPK-like protein kinases [1], [2]. Although the biological function of MAK-V is poorly understood, this protein was recently implicated in the development and progression of mammary gland cancer [3], [4]. However, in another study MAK-V was identified as a suppressor of basal type breast cancers metastasis [5], suggesting cell-type specific mode of MAK-V action. Tumor-promoting properties of MAK-V were linked to anti-apoptotic activity of MAK-V in Her2-overexpressing cells [3]. This is in line with the recently described pro-survival and anti-apoptotic effects of MAK-V in PC12 cells [6]. However molecular mechanisms underlying these effects of MAK-V remain unknown. The only definite association of MAK-V with a particular molecular cascade is the modulating effect of MAK-V on Wnt signaling presumably via MAK-V-directed phosphorylation of Dsh, which was demonstrated in the developing *Xenopus* embryos [7].

To unravel molecular mechanisms of MAK-V action and regulation, and improve our understanding of the role of this protein kinase in cell physiology, we searched for MAK-V-interacting proteins in eukaryotic cells and found that the Nedd4 E3 ubiquitin ligase is able to interact with and affect activity of MAK-V.

## Results

### Identification of Nedd4 as a MAK-V Binding Protein

**Figure 1:**
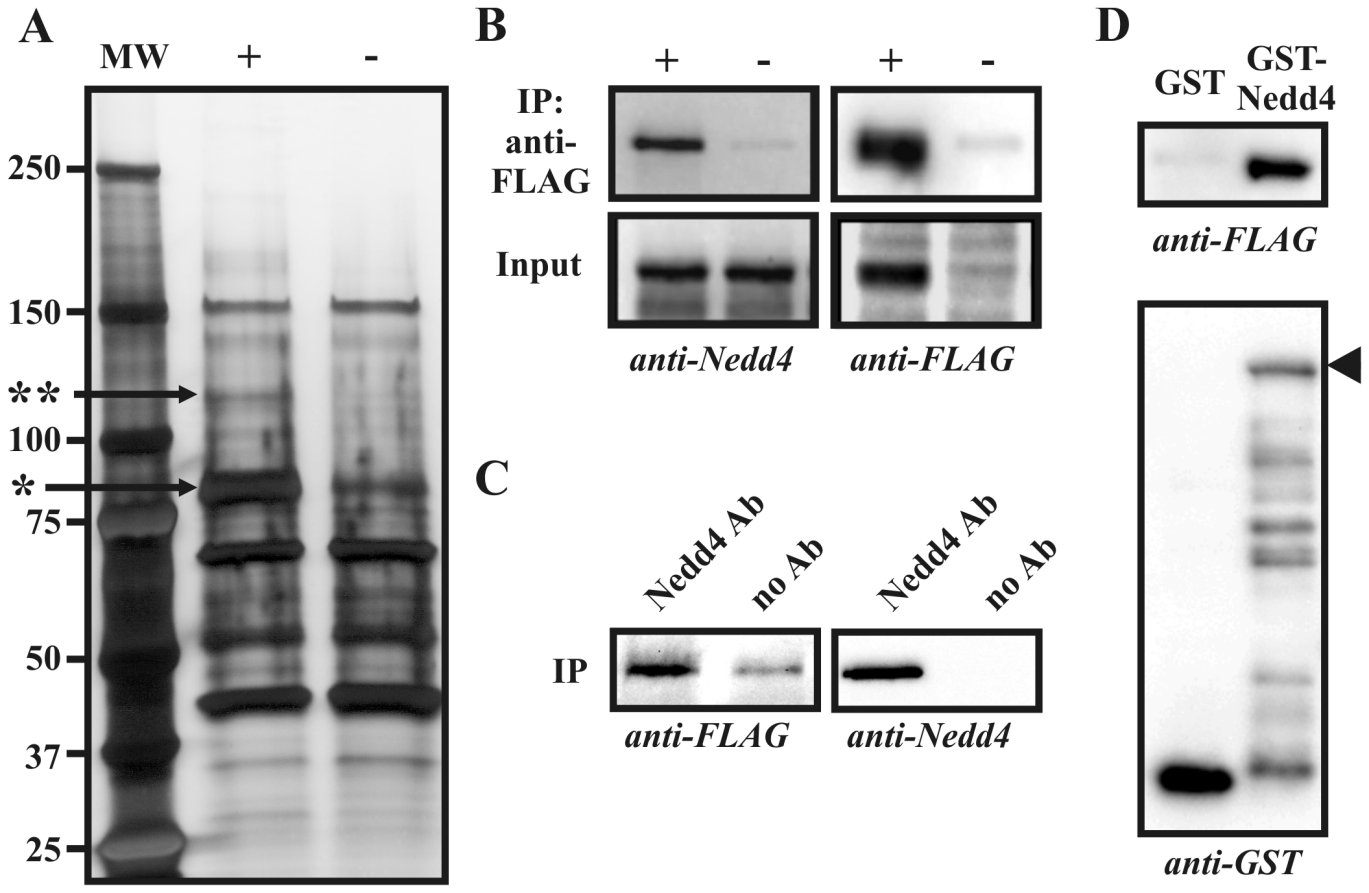
Nedd4 specifically interacts with MAK-V. (**A**) Typical profile of proteins purified on anti-FLAG M2 affinity gel from PC12TetOn cells treated with doxycycline (+) to express MAK-V-FLAG protein or left untreated (-). Silver-stained 4–12% gel is shown with positions of protein molecular weight markers (MW) shown in kD on the left. Arrows marks MAK-V-FLAG protein (*) and Nedd4 protein co-purified with MAK-V-FLAG (**). (**B**) Samples from (**A**) (*IP anti-FLAG*) and aliquots of cell lysates prior to purification (*Input*) were stained with anti-Nedd4 (*anti-Nedd4*) or anti-FLAG (*anti-FLAG*) antibodies. (**C**) Lysate of PC12TetOn cells treated with doxycycline to express MAK-V-FLAG protein was incubated with Protein G Sepharose (*no Ab*) or Protein G Sepharose with immobilized anti-Nedd4 antibodies (*Nedd4 Ab*). Bound proteins were analyzed by Western blotting with anti-FLAG (*anti-FLAG*) or anti-Nedd4 (*anti-Nedd4*) antibodies. (**D**) Proteins were pulled down from lysate of PC12TetOn cells treated with doxycycline to express MAK-V-FLAG protein with GST (*GST*) or GST-Nedd4 (*GST-Nedd4*) proteins immobilized on glutathione Sepharose. MAK-V-FLAG protein was detected with anti-FLAG antibodies (*anti-FLAG*). To monitor GST protein loading of glutathione Sepharose, eluted proteins were stained with anti-GST antibodies (*anti-GST*). Arrowhead marks full-length GST-Nedd4 chimeric protein.

To identify proteins that form complexes with MAK-V *in vivo* we used PC12 cells with doxycycline inducible expression of C-terminally FLAG-tagged MAK-V protein. Lysates of DOX-induced and non-induced cells were loaded on anti-FLAG affinity gel, and following thorough washes bound proteins were eluted and resolved in SDS-PAGE to identify proteins co-purifying with MAK-V. Comparison of eluted protein profiles from induced and non-induced cells allowed us to distinguish between proteins interacting with MAK-V from those absorbing non-specifically on the affinity matrix. A prominent band of ∼115 kD evident only in samples purified from lysates of MAK-V-expressing cells (Fig. 1A) was excised from the gel and subjected to trypsinization and MALDI-TOF mass-spectrometry. Peptide fingerprint analysis identified this protein as rat E3 ubiquitin ligase Nedd4. To confirm the results of mass-spectrometry analysis and the specificity of interaction, we immunoblotted anti-FLAG immunoprecipitates with specific anti-Nedd4 antibodies. This demonstrated that Nedd4 protein indeed specifically co-precipitated with MAK-V protein kinase (Fig. 1B). Reciprocal immunoprecipitation with anti-Nedd4 antibodies also confirmed the existence of MAK-V/Nedd4 complexes in cell lysates although some non-specific MAK-V-FLAG protein absorption on the protein G matrix used to immobilize anti-Nedd4 antibodies was observed (Fig. 1C). To further prove that MAK-V specifically interacts with Nedd4, lysates of MAK-V-FLAG expressing cells were incubated with GST or GST-Nedd4 fusion protein immobilized on glutathione Sepharose. As evident from Fig. 1D, GST-Nedd4 protein and not GST is able to specifically pull down MAK-V-FLAG protein. Together these data demonstrate that MAK-V protein kinase and Nedd4 E3 ubiquitin ligase form specific complexes in cells.

**Figure 2:**
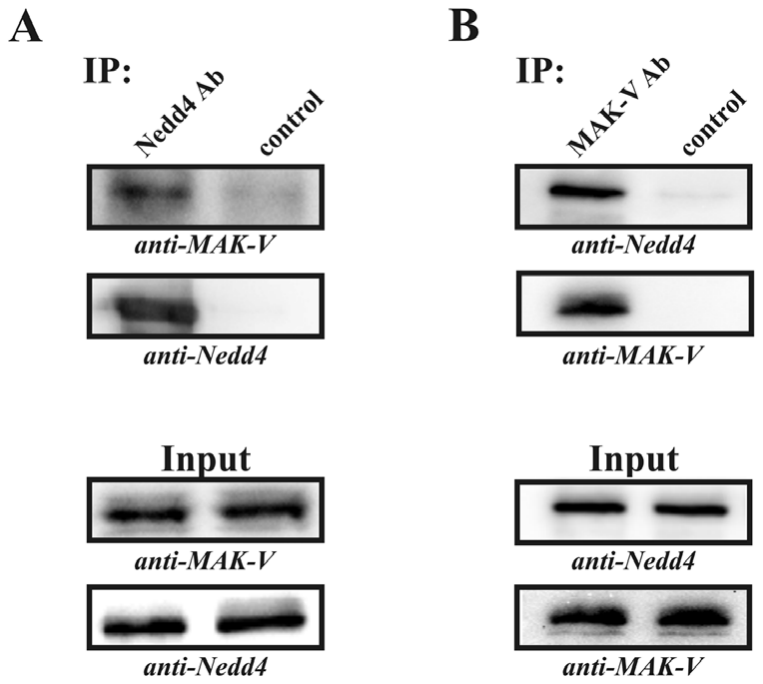
Interaction between endogenous Nedd4 and MAK-V proteins. Proteins were immunoprecipitated from lysates of CSML-0 cells with anti-Nedd4 (**A**; *IP: Nedd4 Ab*) or anti-MAK-V antibodies (**B**; *IP: MAK-V Ab*). Control immunoprecipitations with omitted antibodies were run in parallel (*IP: control*). Immunoprecipitates and aliquots of lysates used for immunoprecipitation (*Input*) were probed with anti-MAK-V (*anti-MAK-V*) and anti-Nedd4 (*anti-Nedd4*) antibodies to detect MAK-V and Nedd4 proteins, respectively.

Although these results unambiguously demonstrate specific interaction between MAK-V and Nedd4, they were obtained in experiments that analyzed interaction between endogenous or recombinant Nedd4 and exogenously produced MAK-V. To demonstrate interaction of endogenous MAK-V and Nedd4 proteins, we assayed the presence of MAK-V/Nedd4 complexes in CSML-0 cells, which produce detectable amounts of endogenous MAK-V protein [8], using co-immunoprecipitation approach. The CSML-0 cell lysate was incubated with Protein G Sepharose beads (control immunoprecipitation) or Protein G Sepharose beads conjugated with anti-Nedd4 antibodies and immunoprecipitates were analyzed for the presence of MAK-V. As evident from Fig. 2A, endogenous MAK-V protein is readily detected in a fraction of proteins immunoprecipitated with anti-Nedd4 antibodies but not in the control immunoprecipitate. To further confirm the interaction between endogenous MAK-V and Nedd4 proteins, we carried out reciprocal immunoprecipitation experiments using anti-MAK-V antibodies. While Nedd4 protein was readily detected in a fraction of proteins immunoprecipitated with anti-MAK-V antibodies, virtually no Nedd4 protein was present in control immunoprecipitates with anti-MAK-V antibodies omitted (Fig. 2B). Together results of immunoprecipitation experiments indicate that endogenous Nedd4 and MAK-V proteins interact with each other.

Nedd4 belongs to a group of structurally related HECT-type E3 ubiquitin ligases with Nedd4-2 being the most closely related to Nedd4 [9], [10]. However, unlike Nedd4, we failed to detect specific enrichment of the Nedd4-2 protein in anti-FLAG immunoprecipitates from lysates of MAK-V-FLAG expressing cells (data not shown). This observation indicates that interaction with MAK-V is specific for Nedd4 but not for Nedd4-2 E3 ubiquitin ligase despite their high similarity.

### Mapping Interaction between MAK-V and Nedd4

**Figure 3:**
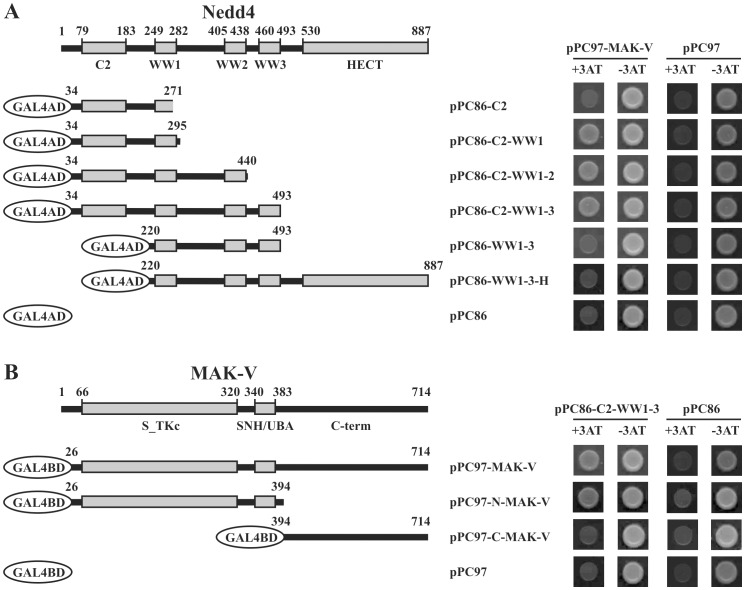
Mapping of interaction between Nedd4 and MAK-V proteins in yeast two-hybrid system. (**A**) Domain structure of mouse Nedd4 protein showing positions (in aa) of C2, WW and HECT domains. The scheme below illustrates organization of GAL4AD proteins fused to various fragments of Nedd4, which were used in the yeast two-hybrid assay. On the right, results of analysis of interaction between GAL4BD fused to MAK-V protein (*pPC97-MAK-V*) and GAL4AD fused to various fragments of Nedd4 are shown. Protein interaction was monitored by ability of yeast to grow in the presence of 3AT (*+3AT*). To monitor specificity, interaction with GAL4BD alone (*pPC97*) was monitored in the same assay. (**B**) Domain structure of mouse MAK-V protein kinase showing positions (in aa) of catalytic (*S_TKc*) and SNH/UBA domains and C-terminal region (*C-term*). The scheme below illustrates organization of GAL4BD proteins fused to full-length of MAK-V or its N- or C-terminal fragments, which were used in the yeast two-hybrid assay. On the right, results of analysis of interaction between GAL4AD fused to fragment encompassing C2 and tree WW domains of Nedd4 (*pPC86-C2-WW1-3*) and GAL4AD fused to MAK-V fragments are shown. Protein interaction and specificity were monitored as described above.

Domain organization of rodent Nedd4 is typical for Nedd4-like HECT-type E3 ubiquitin ligases with a C2 domain on the N-terminus followed by three WW domains and a C-terminally located catalytic HECT domain [11] (Fig. 3A). The interaction of HECT ligases with other proteins is usually mediated by WW domains via proline-rich PY-motifs on their interacting partners [9], [10], [12]. However MAK-V lacks any consensus PY-motif, which suggests a non-canonical mechanism of molecular interaction with Nedd4. Indeed, analysis of interaction between MAK-V and various Nedd4 deletion mutants in the yeast two-hybrid system showed that C2 and the first WW1 domain are sufficient to mediate MAK-V binding while WW domains alone failed to interact with MAK-V (Fig. 3A). Reciprocal experiments with deletion mutants of MAK-V demonstrated that the N-terminal region of the MAK-V polypeptide chain containing protein kinase catalytic and SNH/UBA domains but not a unique C-terminal region, provides interaction interface with Nedd4 (Fig. 3B). This part of MAK-V is also involved in the interaction with all but one [13] known binding partners of this protein.

### MAK-V is Subjected to Proteasome-dependent Degradation

**Figure 4:**
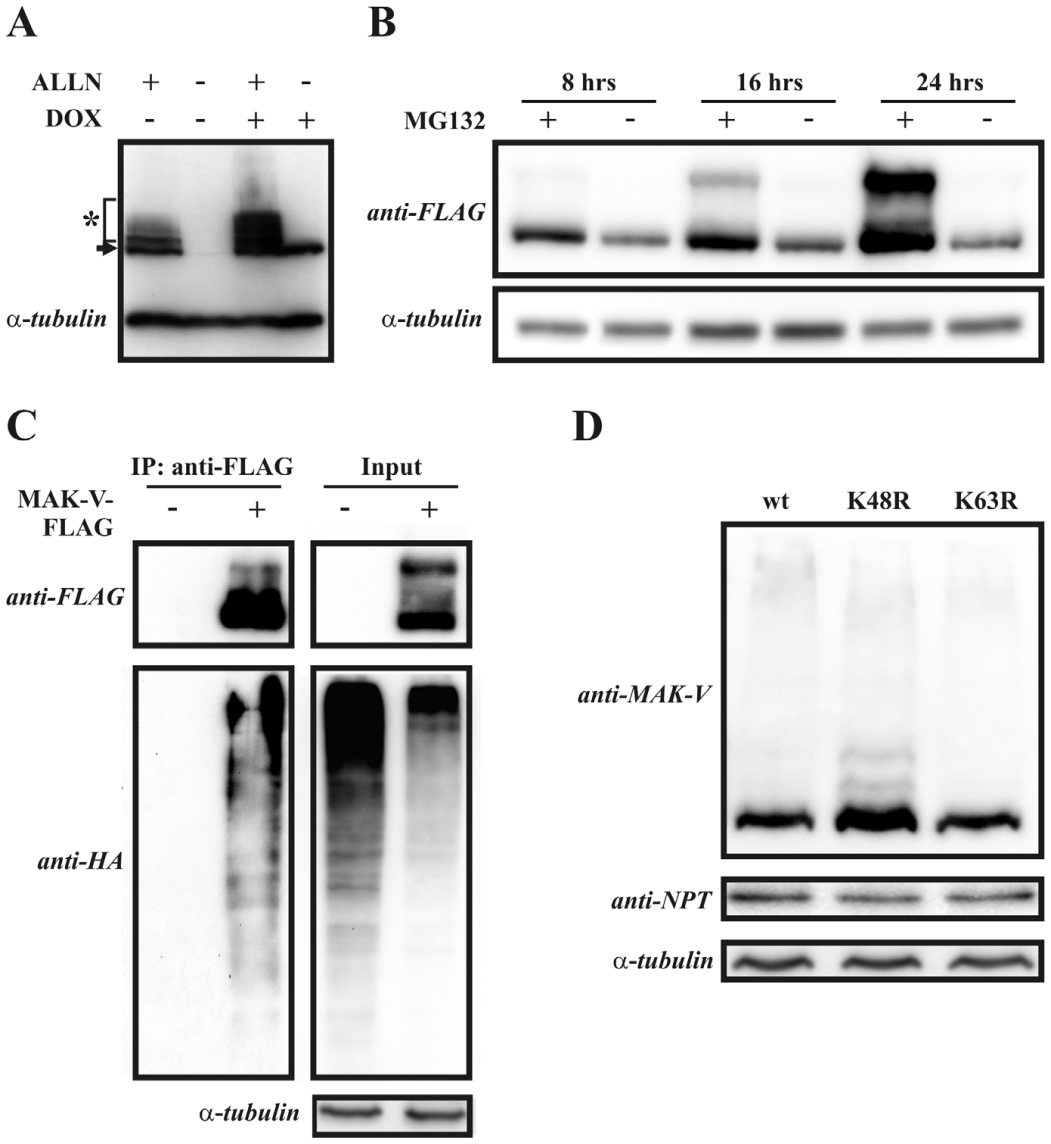
MAK-V is subjected to ubiquitin-dependent proteasomal degradation. (**A**) Doxycycline-treated (*DOX +*) or untreated (*DOX* -) PC12TetOn MAK-V-FLAG cells were incubated with 100 µM of ALLN (*ALLN* +) or vehicle (*ALLN* -) for 8 hrs. Results of Western blot analysis of total cell lysates with anti-FLAG antibodies are shown. MAK-V-FLAG protein marked with arrow, ubiquitinated higher molecular weight MAK-V-FLAG species marked by asterisk. To monitor total protein loading, membrane was re- probed with anti-α-tubulin antibodies. (**B**) HEK293 cells were transiently transfected with plasmid for MAK-V-FLAG protein expression and treated with 10 µM MG132 (*MG+*) for indicated time prior to lysis or left untreated (*MG-*). Lysates were blotted with anti-FLAG antibodies to detect MAK-V-FLAG protein (*anti-FLAG*). To monitor total protein loading, membrane was re-probed with anti-α-tubulin antibodies. (**C**) HEK293 cells were transfected with plasmid for HA-tagged ubiquitin expression alone (*MAK-V-FLAG -*) or together with plasmid for MAK-V-FLAG protein expression (*MAK-V-FLAG +*). Cells were treated with MG132 prior to lysis. MAK-V-FLAG protein was precipitated from lysates with anti-FLAG M2 affinity gel as described for PC12TetOn cells. Anti-FLAG antibodies were used to detect MAK-V-FLAG protein (*anti-FLAG*) in immunoprecipitates (*IP: anti-FLAG*), and ubiquitin was detected with anti-HA antibodies (*anti-HA*). To monitor HA-tagged ubiquitin and MAK-V-FLAG protein expression, aliquots of lysates were blotted as described above. To monitor total protein loading, membrane was re-probed with anti-α-tubulin antibodies. (**D**) HEK293 cells were co-transfected with MAK-V-FLAG expression plasmid and plasmid for expression of FLAG-tagged wild-type ubiquitin (*wt*), its K48R (*K48R*) or K63R (*K63R*) mutant. Results of Western blot analysis of total cell lysates with anti-MAK-V antibodies are shown. To monitor total protein loading, membrane was re-probed with anti-α-tubulin antibodies. To control variations in basal level of MAK-V-FLAG protein expression, samples were also probed with antibodies against neomycin phosphotransferase II (*anti-NPT*) which is encoded by MAK-V-FLAG expression vector.

The identified physical interaction between MAK-V and Nedd4 suggests that this E3 ubiquitin ligase might target MAK-V for ubiquitin-dependent degradation. Noteworthy, MAK-V has been previously suggested to be ubiquitinated [14]. Therefore we assessed whether MAK-V undergoes proteasome-dependent degradation in cells. Treatment of MAK-V-FLAG expressing PC12 cells with the proteasome inhibitor ALLN resulted in an increase in MAK-V-FLAG protein content concomitant with appearance of anti-FLAG reactive protein species of higher molecular weights, which is typical for ubiquitinated proteins targeted to degradation upon proteasome inhibitor treatment (Fig. 4A). This result indicates that MAK-V is indeed targeted to proteasome-dependent degradation. Similar alterations in the pattern of anti-FLAG immunoreactivity were observed following the treatment of HEK293 cells transiently expressing MAK-V-FLAG protein with MG132 proteasome inhibitor (Fig. 4B). Increase in MAK-V protein content in both PC12TetOn and HEK293 cells upon proteasome function inhibition suggests that the control of MAK-V abundance in cells by proteasome-dependent degradation is not a cell type-specific mechanism.

Although MAK-V was previously revealed as an ubiquitinated protein in proteome-wide analysis [14], there is no direct evidence of MAK-V ubiquitination. To directly demonstrate that MAK-V is modified by ubiquitin, HEK293 cells were co-transfected with a HA-tagged ubiquitin expression plasmid alone or together with a plasmid encoding MAK-V-FLAG protein. After treatment with MG132, proteins were precipitated from cell lysates with anti-FLAG antibodies and the presence of ubiquitinated proteins in immunoprecipitates was analyzed by immunoblotting with anti-HA antibodies. As evident from Fig. 4C, ubiquitinated proteins are detected only if MAK-V-FLAG protein was present in cell lysate used for anti-FLAG immunoprecipitation. This suggests that the ubiquitinated protein is MAK-V protein kinase. Moreover, co-transfection of HEK293 cells with MAK-V-FLAG expression plasmid and a plasmid encoding wild-type ubiquitin or its K48R or K63R mutant resulted in a marked increase of MAK-V-FLAG protein only when co-expressed with ubiquitin bearing K48R mutation, which is deficient in polyubiquitin chain formation (Fig. 4D). As K48R ubiquitin mutant expression has a dominant effect on the degradation of ubiquitinated proteins (see, for example, [15], [16]), these results indicate that the MAK-V protein is degraded in an ubiquitin-dependent manner. Together, our data provide evidence of ubiquitin-dependent degradation of MAK-V protein kinase in cells.

### Nedd4 is not Involved in MAK-V Degradation

**Figure 5:**
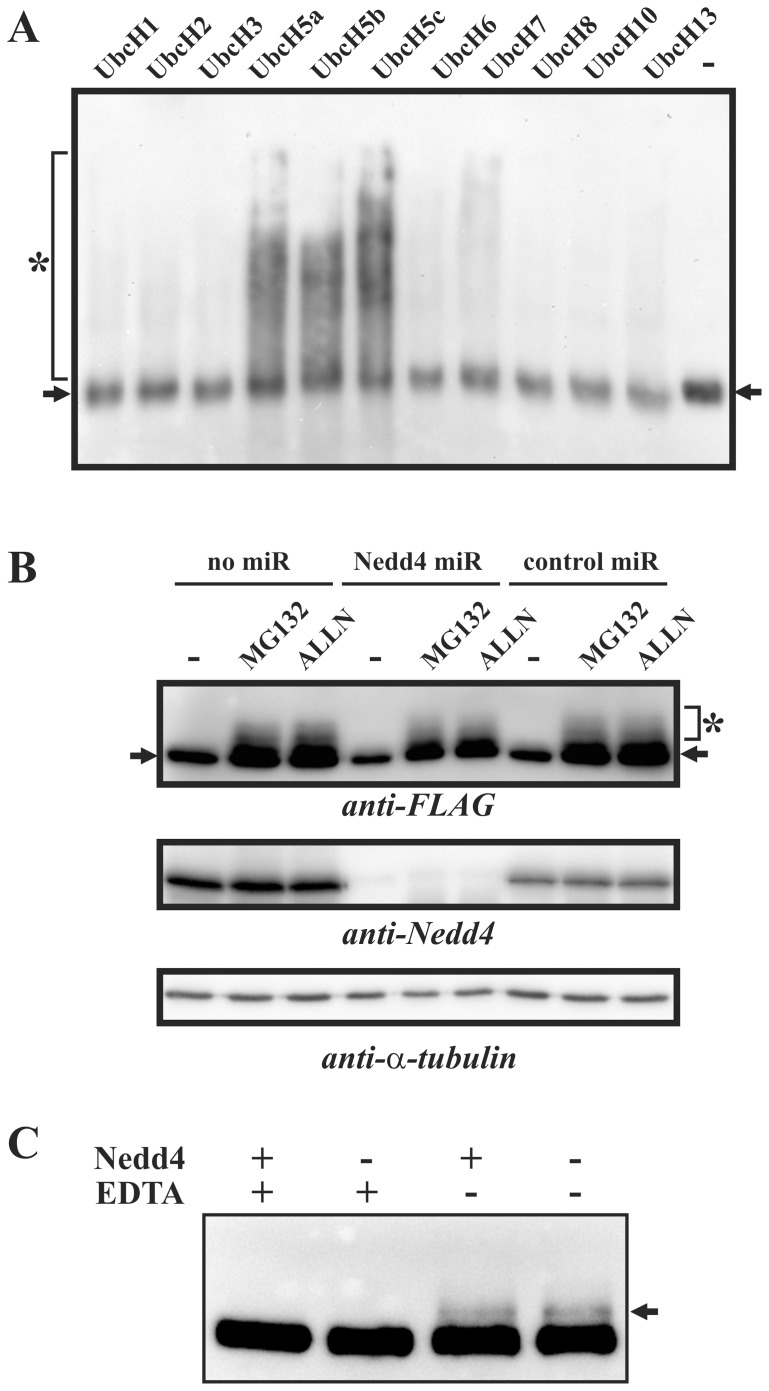
MAK-V is not ubiquitinated by Nedd4. (**A**) E2 enzyme specificity of ubiquitin-ligase activity co-purified with MAK-V-FLAG protein. MAK-V-FLAG protein purified from PC12TetOn cells was used in *in vitro* ubiquitination reactions containing E1 enzyme and indicated E2 ubiquitin-conjugating enzymes. Results of Western blotting with HRP-conjugated streptavidin to detect ubiquitinated proteins/polyubiquitin (marked by asterisk) are shown. The image was overlaid with an image of the same membrane that was consequently probed with anti-FLAG antibodies to detect MAK-V-FLAG protein (marked by arrows). (**B**) Depletion of Nedd4 does not affect stabilization of MAK-V-FLAG protein in cells in response to treatment with proteasome inhibitors. Parental PC12TetOn cells with inducible MAK-V FLAG expression (*no miR*) or their clonal derivatives expressing control microRNA (*control mi*R) or microRNA to target Nedd4 transcript (*Nedd4 miR*) were treated with doxycycline to express MAK-V-FLAG protein and incubated with 100 µM of ALLN (*ALLN*) or 10 µM of MG132 (*MG132*) for 8 hrs or left untreated (-). Results of Western blot analysis of total cell lysates with anti-FLAG antibodies are shown (*anti-FLAG*). Nedd4 level was monitored with anti-Nedd4 antibodies (*anti-Nedd4*). MAK-V-FLAG protein marked with arrow, ubiquitinated higher molecular weight MAK-V-FLAG species marked by asterisk. Membrane was also probed with anti-α-tubulin antibodies (*anti-α-tubulin*) to monitor total protein loading. (**C**) MAK-V-FLAG protein and cytosols were prepared from parental PC12TetOn cells with inducible MAK-V-FLAG expression (*Nedd4*+) or its Nedd4-depleted clonal derivative expressing Nedd4 microRNA (*Nedd4* -) and used in *in vitro* ubiquitination reaction containing E1 and UbcH5b E2 enzymes. Results of Western blot analysis with anti-FLAG antibodies are shown. Arrow marks products of MAK-V ubiquitination. Reactions in the presence of EDTA (*EDTA* +) were used as negative controls.

Next we questioned if Nedd4 was an E3 ubiquitin ligase for MAK-V. When MAK-V-FLAG protein purified from PC12 cells was used in *in vitro* ubiquitination assay with different E2 ubiquitin-conjugating enzymes, a pronounced ubiquitin smear starting from the MAK-V protein band position was evident for Ubc5a, 5b and 5c E2 proteins but not for other E2 proteins assayed (Fig. 5A). The observed E2 enzyme specificity of polyubiquitin chain formation well fit to the E2 protein preference for Nedd4 [17], which is present in MAK-V-FLAG protein preparation used (see Fig. 1). However we failed to detect anti-MAK-V or anti-FLAG reactive species with a molecular weight higher than that of unmodified MAK-V-FLAG protein in *in vitro* ubiquitination reaction products (data not shown), which contradicts the idea that the observed ubiquitin smears represent ubiquitinated MAK-V-FLAG protein. We also attempted to reconstitute *in vitro* MAK-V protein ubiquitination by Nedd4 using recombinant Nedd4 and MAK-V proteins either produced by *in vitro* translation, or expressed in *E.coli*, or purified from transfected mammalian cells. However we repeatedly failed to observe MAK-V modification by ubiquitin (data not shown), which further suggests that MAK-V is not a Nedd4 substrate for ubiquitination. To ultimately prove this, we assayed whether Nedd4 is dispensable for MAK-V destabilization in PC12 cells. We generated clones of MAK-V-FLAG producing cells with expression of microRNA to deplete Nedd4 protein or with control microRNA, and assayed if depletion of Nedd4 would result in the loss of proteasome inhibitor-dependent stabilization of MAK-V-FLAG protein. Expression of Nedd4 microRNA resulted in significant reduction of Nedd4 protein levels while control microRNA had no effect (Fig. 5B). Treatment with both MG132 and ALLN proteasome inhibitors led to similar stabilization of MAK-V-FLAG protein independently of Nedd4 presence in cells (Fig. 5B), suggesting that depletion of Nedd4 does not affect MAK-V stability. Consistently with the suggestion that Nedd4 is not able to ubiquitinate MAK-V, a higher molecular weight anti-FLAG reactive band attributed to MAK-V ubiquitination was observed in *in vitro* ubiquitination reaction products with MAK-V-FLAG and cytosolic proteins derived from Nedd4 depleted cells (Fig. 5C). Taken together, our data strongly suggest that MAK-V is not a target for ubiquitination by Nedd4.

**Figure 6:**
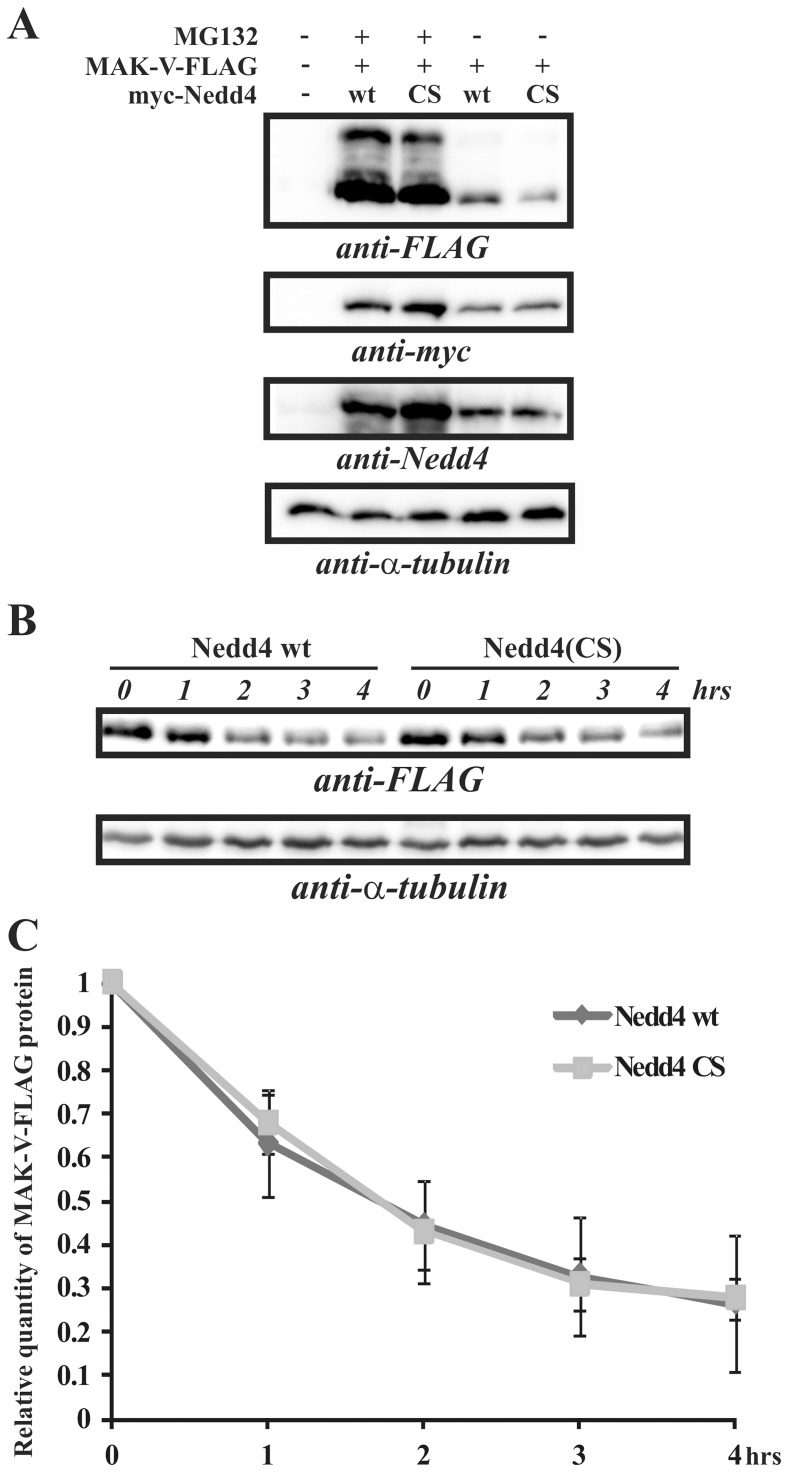
Nedd4 does not affect MAK-V protein level. (**A**) HEK293 cells were transfected with MAK-V-FLAG expression plasmid (*MAK-V-FLAG +*) together with plasmid for expression of *myc*-tagged wild-type Nedd4 (*wt*) or its catalytically inactive Nedd4(CS) mutant (*CS*), or mock-untransfected (-). Cells were treated by MG132 prior to lysis (*MG132+*) or left untreated (*MG132 -*). Results of Western blot analysis of total cell lysates with anti-FLAG antibodies are shown (*anti-FLAG*). Nedd4 levels were monitored with anti-Nedd4 antibodies (*anti-Nedd4*), and exogenously expressed myc-tagged Nedd4 proteins were detected with anti-*myc* antibodies (*anti-myc*). To monitor total protein loading, membrane was re-probed with anti-α-tubulin antibodies. (**B**) HEK293 cells were co-transfected with MAK-V-FLAG expression plasmid and plasmid for expression of *myc*-tagged wild-type Nedd4 (*Nedd4 wt*) or its catalytically inactive Nedd4(CS) mutant (*Nedd4(CS*)). Cells were treated with cycloheximide for indicated time prior to lysis. Representative results of Western blot analysis of total cell lysates with anti-FLAG antibodies to detect MAK-V-FLAG protein are shown (*anti-FLAG*). To monitor total protein loading, membrane was re-probed with anti-α-tubulin antibodies. (**C**) Quantification of MAK-V-FLAG protein level in HEK293 cells when co-expressed with wild-type Nedd4 (*Nedd4 wt*) or with its Nedd4(CS) (*Nedd4 CS*) mutant after cycloheximide treatment. Relative MAK-V-FLAG protein levels for each time point of cycloheximide treatment indicated in hours (*hrs*) were determined by quantification of Western blot images obtained as described in (**B**). Data are presented as means ± standard deviation of three independent experiments performed as described in (**B**).

Although MAK-V failed to interact with Nedd4-2, an ubiqutin ligase most closely structurally related to Nedd4, it cannot be excluded that E3 ubiquitin ligase redundancy in cells may obscure the effect of Nedd4 depletion on MAK-V protein stability. To resolve this issue, the effect of Nedd4 overexpression on MAK-V stability was assessed. As PC12TetOn cells express high levels of endogenous Nedd4 protein, we used HEK293 cells expressing a significantly lower level of this protein (data not shown). Overexpression of Nedd4 should result in the down-regulation of MAK-V protein steady-state level and decreased the protein half-life, provided that Nedd4 is a E3 ubiquitin ligase for MAK-V. As a reference control, we used a catalytically inactive Nedd4(CS) mutant which might also act in a dominant-negative mode through target protein sequestration and thus preventing them from being ubiquitinated and subsequently degraded. However, we failed to reveal any difference in the steady-state levels of MAK-V-FLAG protein transiently expressed in HEK293 cells together with wild-type or mutant Nedd4 (Fig. 6A). Accordingly, no differences were observed in kinetics of MAK-V protein decay after *de novo* protein synthesis was blocked by cycloheximide (Fig. 6B,C). Together, these results demonstrate that Nedd4 is not a MAK-V E3 ubiquitin ligase and does not regulate MAK-V protein stability in cells.

### Nedd4 Affects MAK-V Functions Independently of E3 Ubiquitin Ligase Activity

**Figure 7:**
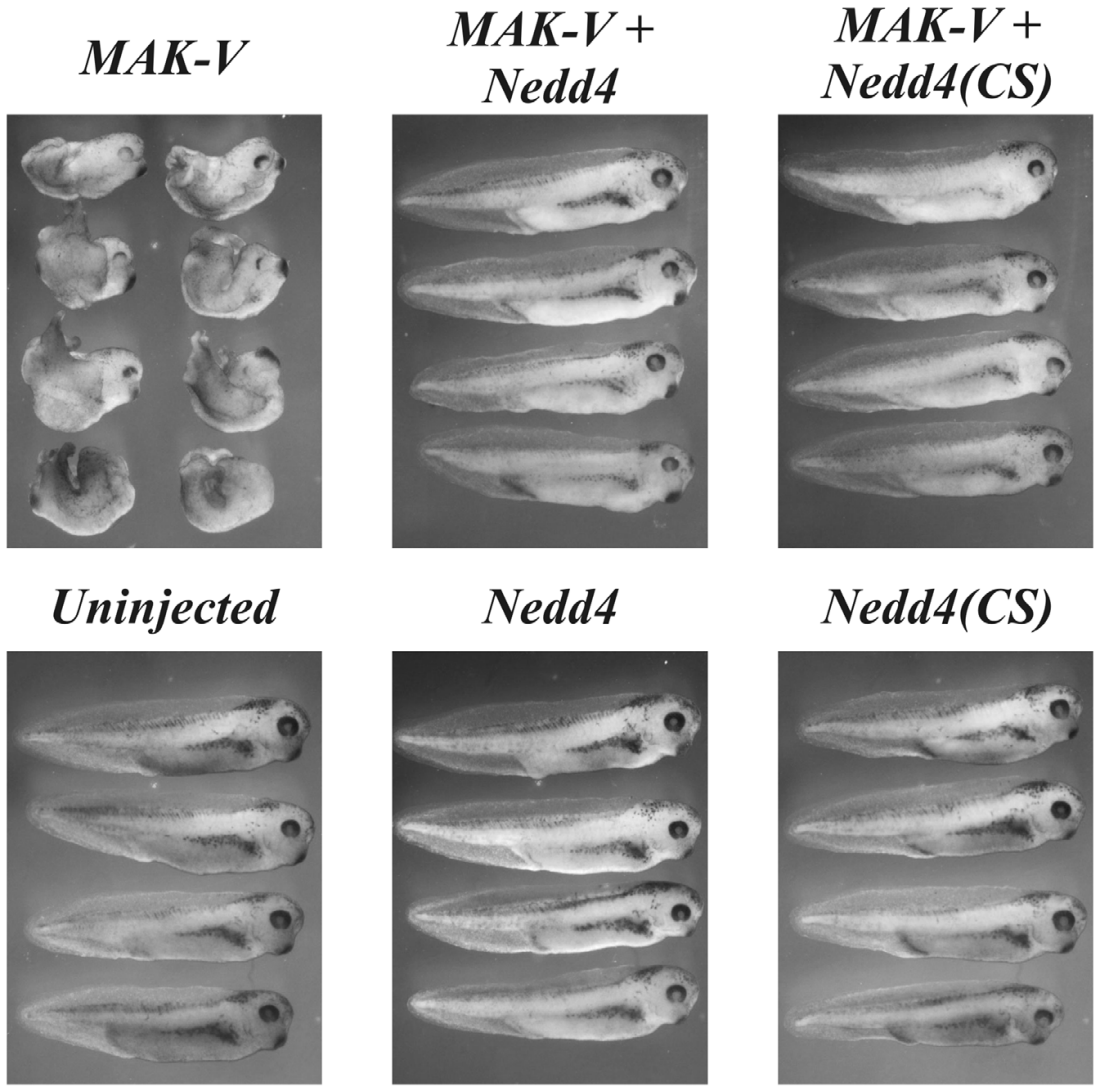
Nedd4 regulates MAK-V activity. Nedd4 in ubiquitin ligase-independent manner rescue axis extension defects in *Xenopus* development induced by MAK-V overexpression. Two ng each of *MAK-V*, *Nedd4*, *Nedd4(CS)* mRNA was injected alone or together as indicated into two dorso-animal blastomeres at 4–8 cell stage. Embryos were fixed at stage 38.

It has been shown previously that overexpression of MAK-V in *Xenopus* embryos inhibits convergent extension movements through the regulation of Wnt signaling [7]. We used this system to investigate if Nedd4 can affect MAK-V function. Consistent with the previous report [7], injection of 1–2 ng of *MAK-V* mRNA into two dorso-animal blastomeres at the 4–8 cell stage resulted in strong morphogenetic abnormalities at stage 38 (Fig. 7). Injection of 2 ng of *Nedd4* mRNA or mRNA for expression of catalytically inactive Nedd4(CS) mutant did not cause any gross abnormalities. However co-injection of *Nedd4* and *MAK-V* mRNAs rescued the effect of MAK-V on convergent extension movements. Consistently with the lack of Nedd4-dependent ubiquitination of MAK-V, a similar effect was observed upon Nedd4(CS) expression (Fig. 7). Thus Nedd4 in an ubiquitin ligase-independent manner is capable of suppressing MAK-V-induced developmental defects in the *Xenopus* embryo, thus demonstrating functional interplay between the two proteins and supporting the physiological relevance of the revealed interaction between Nedd4 and MAK-V.

## Discussion

Since its identification and cloning, MAK-V protein kinase has been expected to play a role in tumorigenesis [2], [18]. This has been supported by frequently observed MAK-V overexpression in human breast tumors [19]. Pro-survival and anti-apoptotic properties of this protein kinase revealed in recent studies directly implicated MAK-V in tumor cell biology [3]–[5]. The revealed role of MAK-V in cancer provides a rational basis to consider MAK-V as a target in anti-cancer therapy. However in different types of tumor cells MAK-V expression either promoted [4] or suppressed [5] their metastasis potential. Moreover, in different experimental models MAK-V appeared to be either essential [3] or dispensable [4] for tumorigenesis itself. This apparent contradiction is likely to be caused by specific functional consequences of MAK-V expression within different cellular contexts. In turn, this emphasizes the importance of better understanding of MAK-V molecular mechanisms of action and regulation. This knowledge is required for discriminating between tumors in which MAK-V expression is pro-carcinogenic and those in which MAK-V expression is dispensable for or even suppresses metastasis. Here we revealed two possible modes of MAK-V regulation – control of MAK-V protein abundance through proteasomal degradation and Nedd4-dependent functional suppression of MAK-V.

Identification of interaction partners is a powerful approach for integrating a protein of interest into particular chain of molecular events. Here we identified HECT-type E3 ubiquitin ligase Nedd4 as a most prominent binding partner of MAK-V in PC12 cells and confirmed that two proteins can form a complex when expressed in cells. WW domains of HECT type ubiquitin ligases usually function as interaction interface through binding PY-motifs in a partner protein and also can interact with phosphoserine/phosphothreonine residues, although with lower affinity [9], [10], [12], [20]. In addition, interaction of many proteins with Nedd4 family ubiquitin ligases is known to be mediated by adaptor proteins containing PY-motif capable to bind WW domains of these ubiquitin ligases [10]. The MAK-V protein lacks any consensus PY-motif but WW1 domain of Nedd4 is still required for MAK-V binding. Yet our experimental data did not confirm that WW domains (WW1-WW3) of Nedd4 alone are capable of conferring interaction with MAK-V, which might be explained by low affinity of this interaction. As MAK-V protein is known to be abundantly phosphorylated in cells [21], it becomes feasible that Nedd4 WW domains might secure low affinity binding to phosphorylated serine and/or threonine residue(s) in MAK-V with the additional interaction with the C2 domain of Nedd4 required to stabilize MAK-V/Nedd4 complexes.

Although our data suggested that Nedd4 is not an ubiquitin ligase for MAK-V, Nedd4 was capable of rescuing developmental defects in *Xenopus* embryos caused by MAK-V overexpression. This demonstrates that Nedd4 is a regulator of MAK-V functional activity, ultimately confirming physiological relevance of the identified interaction between MAK-V and Nedd4. The ability of both wild type and ubiquitin ligase-deficient Nedd4 to rescue the MAK-V overexpression phenotype in *Xenopus* embryos indicates that ubiquitin ligase activity is dispensable for Nedd4-mediated regulation of MAK-V.

The simplest model of MAK-V regulation by Nedd4 assumes sequestration of MAK-V protein kinase catalytic activity through interaction with Nedd4. Indeed, Nedd4 binds to the N-terminal part of MAK-V encompassing catalytic and SNH/UBA domains, both of which are required for catalytic activity of related protein kinases [22], [23]. However other scenarios of Nedd4-dependent suppression of MAK-V can be proposed, and the molecular mechanism of MAK-V suppression by Nedd4 is yet to be investigated.

The revealed interaction between MAK-V protein kinase and Nedd4 provide a mechanistic basis for potential regulation of Nedd4 activity by MAK-V phosphorylation. Indeed, functions of Nedd4-like E3 ubiquitin ligases are regulated by phosphorylation, which might either positively or negatively regulate their activities [10]. Thus, it is important to further investigate the role of MAK-V protein kinase in regulation of Nedd4 activity and Nedd4-controlled cellular processes.

Taken together, we found that MAK-V protein kinase is capable of interacting with Nedd4 ubiquitin ligase. Detection of endogenous Nedd4/MAK-V complexes proves that this interaction takes place in physiological conditions. Although molecular mechanisms of mutual effects of these two proteins on each other functions remain to be elucidated, we showed that Nedd4 counteracts effect of MAK-V overexpression on *Xenopus* embryo development. This observation suggests physiological relevance of the identified interaction between MAK-V and Nedd4.

## Materials and Methods

### Plasmids

*Nedd4* cDNA fragment encoding full-length Nedd4 except for the first 33 amino acids (aa 34-887) was excised with BamHI and NotI restriction endonucleases from EST clone IMAGE:6330296 (American Tissue Culture Collection; GeneBank Accession No. BQ885745). The excised fragment was subcloned in-frame into the pBK-CMV plasmid (Stratagene, USA) engineered to contain the fragment of preproinsulin cDNA 5′-untranslated region, ATG codon and sequence encoding *myc* epitope downstream CMV promoter [24]. *Nedd4* cDNA linked to preproinsulin cDNA 5′-untranslated region, the ATG codon and sequence encoding *myc* epitope was then cloned into pSP64(PolyA) vector (Promega, USA) to generate pSP64-Nedd4 plasmid for *Nedd4* mRNA *in vitro* transcription. PCR-directed mutagenesis was used to introduce C854S mutation into *Nedd4* cDNA resulting in catalytically inactive Nedd4(CS) ubiquitine ligase [25]. The presence of introduced substitutions was confirmed by sequencing. pSP64-Nedd4(CS) plasmid for *Nedd4(CS)* mRNA *in vitro* transcription was constructed as described above for pSP64-Nedd4 plasmid.

The pGEX-Nedd4 plasmid for expression of Nedd4 protein (aa 34-887) fused to glutathione-S-transferase (GST) was constructed by cloning the respective *Nedd4* cDNA fragment into pGEX-4T-2 plasmid (GE Healthcare, USA).

Predesigned BLOCK-iT miR RNAi Select hairpin (Invitrogen, USA) to target the rat *Nedd4* transcript was obtained by annealing oligonucleotides 5′-tgc tga taa atc tca ggt cca gct ccg ttt tgg cca ctg act gac gga gct ggc tga gat tta t-3′ (top strand) and 5′-cct gat aaa tct cag cca gct ccg tca gtc agt ggc caa aac gga gct gga cct gag att tat c-3′ (bottom strand) and cloning into pcDNA6.2-GW/EmGFP-miR vector using BLOCK-iT PolII miR RNAi Expression Vector kit (Invitrogen, USA). The resulting pcDNA6.2-GW/EmGFP-miR-rNedd4 plasmid codes for Nedd4 microRNA together with EmGFP fluorescent protein. As a control, pcDNA6.2-GW/EmGFP-miR-neg control plasmid (Invitrogen, USA) for expression of hairpin predicted not to target any known vertebrate gene was used.

Plasmids pPC97 and pPC86 for analysis of protein-protein interaction in yeast were described previously [26], as well as plasmids for expression of DNA-binding domain of GAL4 transcription factor (GAL4BD) fused to mouse MAK-V protein (aa 26-714; pPC97-MAK-V), its N-terminal part (aa 26-394; pPC97-N-MAK-V) or complementary C-terminal portion (aa 395-714; pPC97-C-MAK-V) [27]. To construct plasmids for expression of Nedd4 fragments fused to GAL4 activator domain (GAL4AD) in yeast, respective *Nedd4* cDNA fragments excised with appropriate restriction endonucleases or amplified in PCR were cloned into multiple cloning site of the pPC86 vector. The following plasmids were constructed: pPC86-C2 (Nedd4 aa 34-271 containing C2 domain; see Fig. 3A for domain positions in Nedd4 protein), pPC86-C2-WW1 (Nedd4 aa 34-295 containing C2 and WW1 domains), pPC86-C2-WW1-2 (Nedd4 aa 34-440 containing C2, WW1 and WW2 domains), pPC86-C2-WW1-3 (Nedd4 aa 34-493 containing C2 and all three WW domains), pPC86-WW1-3 (Nedd4 aa 220-493 containing only three WW domains), and pPC86-WW1-3-H (Nedd4 aa 220-887 containing WW1-3 and HECT domains). Details of cloning are available on request.

Plasmids for expression of Ha-tagged ubiquitin [28] and C-terminally FLAG-tagged MAK-V protein kinase [21] were described before. Plasmids for expression of FLAG-tagged ubiquitin and its K48R and K63R mutants were a gift from Dr. Ajay Chitnis (National Institutes of Health, Bethesda, MD, USA).

### Cells

PC12TetOn cells (Clontech, USA) with inducible expression of mouse MAK-V-FLAG protein were cultured as described before [6]. For total cell lysate preparation, cells were plated in 24-well plates (300000 cells per well). If required, cells were treated with 1 µg/ml doxycyline for 2 days prior to lysis. If indicated, cells were treated with 10 µM MG132 (Calbiochem, USA) or 100 µM ALLN (Sigma, USA) prior to lysis.

To generate microRNA-expressing clones, cells were transfected with pcDNA6.2-GW/EmGFP-miR-neg control (Invitrogen, USA) or pcDNA6.2-GW/EmGFP-miR-rNedd4 plasmid and clones were selected in the presence of 2 µg/ml of blasticidin. Homogeneity of the cell population in isolated clones was monitored by EmGFP fluorescence.

CSML-0 cell line established from a mammary adenocarcinoma was described before [29]. Cells were cultured in DME medium supplemented with 10% fetal bovine serum, 100 units/ml of penicillin and 100 µg/ml of streptomycin.

HEK293 cells (European Collection of Cell Cultures, 85120602) were cultured on DME medium supplemented with 10% fetal bovine serum, 100 units/ml of penicillin and 100 µg/ml of streptomycin. Cells were transfected using Unifectin-56 transfection reagent (Unifect Group, Russia). Cells were lysed 2 days after transfection. If indicated, cells were treated with 10 µM MG132 (Calbiochem, USA) for 24 hrs (or 8 and 16 hrs if specified) prior to lysis.

### Isolation of MAK-V-FLAG Protein and MAK-V-interacting Proteins

To isolate MAK-V-FLAG protein and MAK-V-interacting proteins, four 100 mm plates of PC12TetOn cells with inducible MAK-V-FLAG expression were treated with 1 µg/ml of doxicyxline for 2 days to induce MAK-V-FLAG expression or left untreated. Subconfluent cells were washed in ice-cold PBS, collected in microcentrifuge tubes and lysed in 0.5 ml/plate of lysis buffer (1x tris-buffered saline, pH 8.0 (TBS); 1% Nonident P-40; 0.5% Triton X100; 1 mM MgCl_2_; 0.2 mM EGTA; 1x Complete EDTA-free Protease Inhibitor Cocktail (Boehringer Mannheim, Germany), 1x Phosphatase Inhibitor Cocktails I and II (Sigma, USA)) at 4°C for 15 min. Lysates were cleared by centrifugation (4500 g, 10 min, 4°C) and incubated with 50 µl of EZview Red anti-FLAG M2 affinity gel (Sigma, USA) at 4°C for 2 hrs. The gel was washed 3 times with 500 µl of ice-cold lysis buffer and bound proteins were eluted by sequential incubation for 30 min with 50 µl and 25 µl of TBS containing 100 µg/ml of FLAG peptide (Sigma, USA). Eluted proteins were resolved in NuPAGE Bis-Tris 4–12% gel and stained with either SilverXpress Silver Staining Kit (Invitrogen, USA) or Coomassie stain. Protein bands were cut from Coomassie stained gels and trypsinized for further analysis by peptide fingerprint analysis. Mass-spectrometric analysis of protein tryptic digest was done on Ultraflex MALDI-TOF mass spectrometer (Bruker-Daltonics, Germany). Proteins were identified using Mascot program (Matrix Science, USA).

### Cytosol Fraction and Total Cell Lysate Preparation

Cytosol was prepared from PC12TetOn clones as described before [8]. To prepare total cell lysates, cells in wells of 24-well plate were washed with ice-cold PBS and lysed in 120 µl of SDS-PAGE loading buffer. Lysates were boiled prior to loading on the gel. CSML-0 cell lysates for immunoprecipitation were prepared essentially as described for lysate preparation for isolation of MAK-V-FLAG protein but 250 µl of lysis buffer were used to lyse cells from subconfluent 100 mm cell culture dish.

### Western Blot Analysis

Proteins were resolved in SDS-PAGE and transferred to Hybond P membrane (GE Healthcare, Little Chalfont, UK). Membranes were incubated with primary rabbit polyclonal anti-FLAG (Sigma, USA), anti-Nedd4 (Abcam, UK), anti-Nedd4-2 [30], or anti-neomycin phosphotransferase II (Millipore, USA), mouse monoclonal anti-α-tubulin, clone DM1A antibodies (Sigma, USA) or affinity purified rat anti-MAK-V antibodies [8] followed by secondary anti-rabbit, anti-mouse or anti-rat horseradish peroxidase-conjugated antibodies (HRP; GE Healthcare, USA) as described previously [31]. To detect GST, anti-GST HPR conjugate (GE Healthcare, USA) was used. Biotinylated proteins were detected with HRP-conjugated streptavidin (Calbiochem, USA). Immobilon Western Chemiluminescent HRP Substrate (Millipore, Billerica, MA, USA) or ECL+ reagent (GE Healthcare, USA) were used for detection. Images were acquired on ChemiDoc XRS gel documentation system (BioRad, Hercules, CA, USA).

### Quantification of MAK-V-FLAG Protein Level in Cycloheximide-treated Cells

HEK293 were treated with 10 µM cycloheximide (Fluka, Switzerland) for 0, 1, 2, 3 or 4 hrs prior to lysis in SDS-PAGE loading buffer. Lysates were first immunoblotted with anti-FLAG antibodies, and after washing the blot was re-probed with anti-α-tubulin antibodies. Chemiluminescence was detected with ChemiDoc XRS gel documentation system (BioRad) and quantified with QuantityOne 4.6.2 software (BioRad). The levels of MAK-V-FLAG protein for each time point were first normalized to α-tubulin level, and then expressed as percentage of the MAK-V-FLAG protein level at 0 hr point taken as 1.00. Each experiment was repeated three times, and percentage of MAK-V-FLAG protein levels for each time point was calculated as average in three experiments ± standard deviation.

### *In vitro* Ubiquitination Assay

*In vitro* ubiquitination with biotinylated ubiquitin was performed in 20 µl reactions using Ubiquitinylation kit (Biomol, USA) according to supplier’s instructions. Seventy (if cytosol was added to reaction) or 130 ng (if cytosol was omitted) of purified MAK-V-FLAG protein and 9.6 µg of cytosolic proteins, if included, were used in each reaction. Reaction products were analyzed by Western blotting with either protein specific antibodies or total ubiquitin was detected with HRP-conjugated streptavidin.

### GST Pull-down Assay

GST and GST-Nedd4 proteins were expressed in Rozetta-gammi *E.coli* (Novagen, USA) transformed with pGEX-4T-2 and pGEX-Nedd4 plasmids, respectively, and purified on glutathione Sepharose (GE Healthcare, USA) according to manufacturer’s recommendations. Eluted proteins were dialyzed against PBS and stored at −70°C. Glutathione Sepharose beads (20 µl) were incubated with 30 µg of GST or GST-Nedd4 protein for 1 hr at 4°C with rotation and washed to remove unbound protein. Beads were blocked with 0.2 µm-filtered 3% membrane blocking agent (GE Healthcare, USA) in TBS for 30 min at 4°C, washed in TBS and incubated for 2 hrs at 4°C with 50 µl of MAK-V-FLAG expressing cell lysate prepared as described above for MAK-V-FLAG protein purification. Beads were washed with lysis buffer and bound proteins were eluted by boiling in SDS-PAGE sample buffer. Eluted proteins were analyzed by Western blotting with anti-FLAG antibodies to detect MAK-V-FLAG protein, and with anti-GST antibodies to monitor GST protein loading.

### Immunoprecipitation

Five hundred µl of CSML-0 cell lysate were incubated overnight in SigmaPrep Spin column (Sigma, USA) at 4°C with 2 µg of anti-Nedd4 antibodies (Abcam, UK), 4 µg of anti-MAK-V antibodies [8], or without antibodies added (control immunoprecipitations). After that, 20 µl of Protein G Sepharose beads (GE Healthcare, USA) were added, and incubation was continued with rotation for 4 hrs at 4°C. Beads were washed 3 times with ice-cold buffer used to prepare cell lysates, and transferred as a suspension to Eppendorf tube. After brief centrifugation, supernatant was aspirated with 30G needle, and 20 µl of SDS-PAGE loading buffer were added to beads to elute proteins. Samples were boiled prior to loading on the gel. For control, immunoprecipitation samples were processed in a similar way but antibodies were omitted in primary incubation with Protein G Sepharose.

### Protein Interactions in Yeast Two-hybrid System

Analysis of protein interactions in yeast was done essentially as described before [1] using yeast expressing assayed proteins as chimeras with GAL4BD and GAL4AD. Interaction was monitored by activation of the *HIS3* reporter gene by the ability of yeast to grow on minimal medium supplemented with 60 mM 3-amino-(1,2,4)-triazole (3AT; Sigma, USA).

### Experiments with *Xenopus* Embryos

Synthesis of mouse *MAK-V* RNA for injection, embryo culture and microinjection were done essentially as described before [7]. Mouse *Nedd4* and *Nedd4CS* RNAs for microinjection were synthesized from pSP64-Nedd4 and pSP64-Nedd4(CS) plasmids, respectively, using mMessage mMashine kit (Ambion, USA). One-to-two nanogram of *MAK-V*, *Nedd4*, or *Nedd4(CS)* mRNA were injected alone or together into two dorso-animal blastomeres at 4–8 cell stage, and morphological phenotypes were evaluated at stages 35–38.
